# Adapting the Research Development and Innovation (RD & I) Value Chain in Psychology to Educational Psychology Area

**DOI:** 10.3389/fpsyg.2018.01188

**Published:** 2018-08-24

**Authors:** Jesús de la Fuente, Douglas Kauffman, Unai Díaz-Orueta, Yashu Kauffman

**Affiliations:** ^1^School of Psychology, University of Almería, Almería, Spain; ^2^Facultad de Ciencias Sociales, Universidad Autónoma de Chile, Santiago, Chile; ^3^Independent Educational Researcher, Bedford, MA, United States; ^4^Department of Psychology, Maynooth University, Maynooth, Ireland; ^5^Massachusetts Institute of Technology, Cambridge, MA, United States

**Keywords:** educational psychology, RD & I value chain, RD & I projects, RD & I department, innovation and entrepreneurship projects

## Abstract

Educational Psychology, as an area of Psychology that specializes in formative processes, faces several important challenges in the information and knowledge society of this twenty first century. One of these challenges is to facilitate a paradigm shift from a nearly exclusive focus on social science to the scientific-technological approach of a discipline that produces innovation and meaningful transfer of science and technology. The Research, Development, and Innovation (RD & I) value chain means pursuing these three endeavors in both the academic and professional lines of Educational Psychology. It is a strategy of innovation that leads us to integrate *academic or research* activity (R), research-related or professional *scientific-technological development of innovation* (D) and *transfer and entrepreneurship* activity (I). Generating innovation and transfer, applicable to educational contexts, can be an important stimulus of activity for new practicing psychologists in Educational Psychology. The RD & I value chain can become an academic, research-related or professional advantage in different activities, since it pertains to the processes, products and services found in the sphere of Educational Psychology. Several examples of how the RD & I chain can help improve psychoeducational activities are presented. First, we analyze competitive improvements that the RD & I chain can offer in competitive bids. Second, we give examples of the RD & I chain in the development of new processes, products and services in *Projects of Innovation and Entrepreneurship in Educational Psychology*, specifically illustrating the chain in each case. In order for this conception to take shape, a new cross-functional area must be created in professional and educational organizations. Specifically, this means creating an RD & I Department, or some area that branches across the other functions. The mission of this cross-functional unit is the actual implementation of the RD & I chain in the educational organization, as well as an incentive for innovative activities: use of ICT applications, organizational improvement, improved assessment, analysis of information produced by the organization itself, cost-benefit analysis, strategic decision-making processes, and so on.

## Introduction

Psychology's connection with innovation is inherent in the study and analysis of human behavior. However, a commitment to innovative activity, to knowledge transfer in the sense found in other areas of science/technology, continues to be a pressing need. There are several reasons for this endeavor. Educational Psychology is a discipline that bridges Psychology and Education, and is closely linked to Social and Educational Sciences. The Social Sciences, however, have not traditionally been oriented toward innovation and scientific-technological knowledge. This situation must change if Educational Psychology is to be present among sciences and professions with Information and Communications Technology (ICT)-based innovations, positioned in the educational sphere, just as Psychology is already positioned in other fields of knowledge.

On the one hand, a paradigm shift must be encouraged, moving from the almost exclusive Social Sciences focus to a scientific-technological approach, characteristic of experimental and health-related disciplines that produce meaningful innovation and transfer in today's information and knowledge-based society. On the other hand, new generations of psychologists must begin to engage in experiences and formative processes in the Research & Development & Innovation (RD & I) value chain. The mid- and long-term results would be: (1) Better strategic positioning of the profession and its professionals in the information and knowledge-based society of the twenty first century; (2) Creation of competitive processes, products and services, with high innovative value; (3) New professionals specialized in the RD & I value chain (Voutsinas et al., 2015).

This proposal for innovation continues to be a challenge for academia and for the profession: that the organizations and institutions that carry out the tasks of Educational Psychology would be staffed with new positions based on a new set of professional qualifications.

## Evolution of RD & I value chain in the knowledge society

Educational Psychology, as the area of Psychology that studies formative processes, faces several important challenges in the twenty first century. First, it must help redefine formative processes in the context of an Information and Knowledge-based Society (Punye, 2007). Second, it must encourage reflection on developing competencies of innovation and entrepreneurship in this sphere of academic and professional knowledge. Research, Development and Innovation (RD & I) is a concept that has recently appeared in the context of science, technology and society, replacing the former “Research & Development” (R&D). While “Development” as a term comes from the world of economics, the terms “Research” and “Innovation” come from epistemology and from technology, respectively, and their dynamic relationship is found when differentiating between pure and applied sciences (Cardinal, 2001; Arimura et al., 2007). Each of these terms is complex to define. Aho (2008), provocatively defines “*research*” as investing money in order to obtain knowledge, while “*innovation*” would *be* investing knowledge in order to obtain money, expressing quite well the feedback phenomenon that is produced in a successful RD & I strategy (Edquist, 1997; Boons and Lüdeke-Freund, 2013). When applied in politics and legislation, the concept of RD & I defines (1) *research* as the original, planned inquiry that seeks new knowledge and better understanding in science and technology; (2) *development of technological innovation* as the application of research results, or of any other type of scientific knowledge, for the manufacture of new materials or products and for designing new processes and production systems, as well as for substantial technological improvement in pre-existing materials, products, processes and systems (Bernardino and Freitas, 2017); and (3) *transfer of technological innovation (entrepreneurship)* as the activity that results in technological progress in obtaining new products or production processes, or substantial improvement in those that already exist. Products and processes are considered new if their characteristics or applications, from a technological point of view, differ substantially from those already exinting (Pateli and Giaglis, 2005). Therefore, this sequence of actions has been called the *RD & I value chain* (Sanz and Cruz, 2009).

The level of RD & I activity in a country can be calculated as the ratio between RD & I spending and Gross Domestic Product (GDP), breaking down spending into public and private spending. To the extent that they are able, all countries attempt to encourage RD & I through support policies (subsidies, deductions, soft loans, etc.), since a high level of RD & I means stronger companies, whose products and processes stand out from their competitors'. Furthermore, many such activities can potentially bring about social progress in the form of quality of life, improving the environment, health, and the ecosystem. In order to support these activities, a number of UNE standards exist: the UNE 166000 series, including UNE standard 166001 that addresses RD & I projects, UNE 166002 on requirements for the RD & I management system, and UNE 166006, relative to system requirements for technology watch. This conception is not static, but in constant evolution, and directly affects the tasks and objectives of the Universities and researchers (FECYT, 2011). See Table 1.

**Table 1:** Moments in R&D value chain.

**Moment**	**Context**	**Description**	**Research**	**Technological development**	**Transfer innovation**
1st	Classic	Research as core activity. Research only	A lack of leadership in worldwide research production. Any aim toward technological development is absent	Rarely are technological processes, products or services invented or produced. Few cases of new technological patents or registrations	Limited innovation transfer. Small amounts of scientific-technological entrepreneurship
2nd	Present day	R & D & I	Leadership in worldwide research production. Research based on producing technological developments	Leadership in producing technology processes, products and services. Leadership in patent production	Innovation transfer as a purpose for research. Leadership in scientific-technological entrepreneurship

## Professional implications of the different conceptions of research, technological development and innovation in academics

### Consequences of the classical value chain

**Figure 1:**
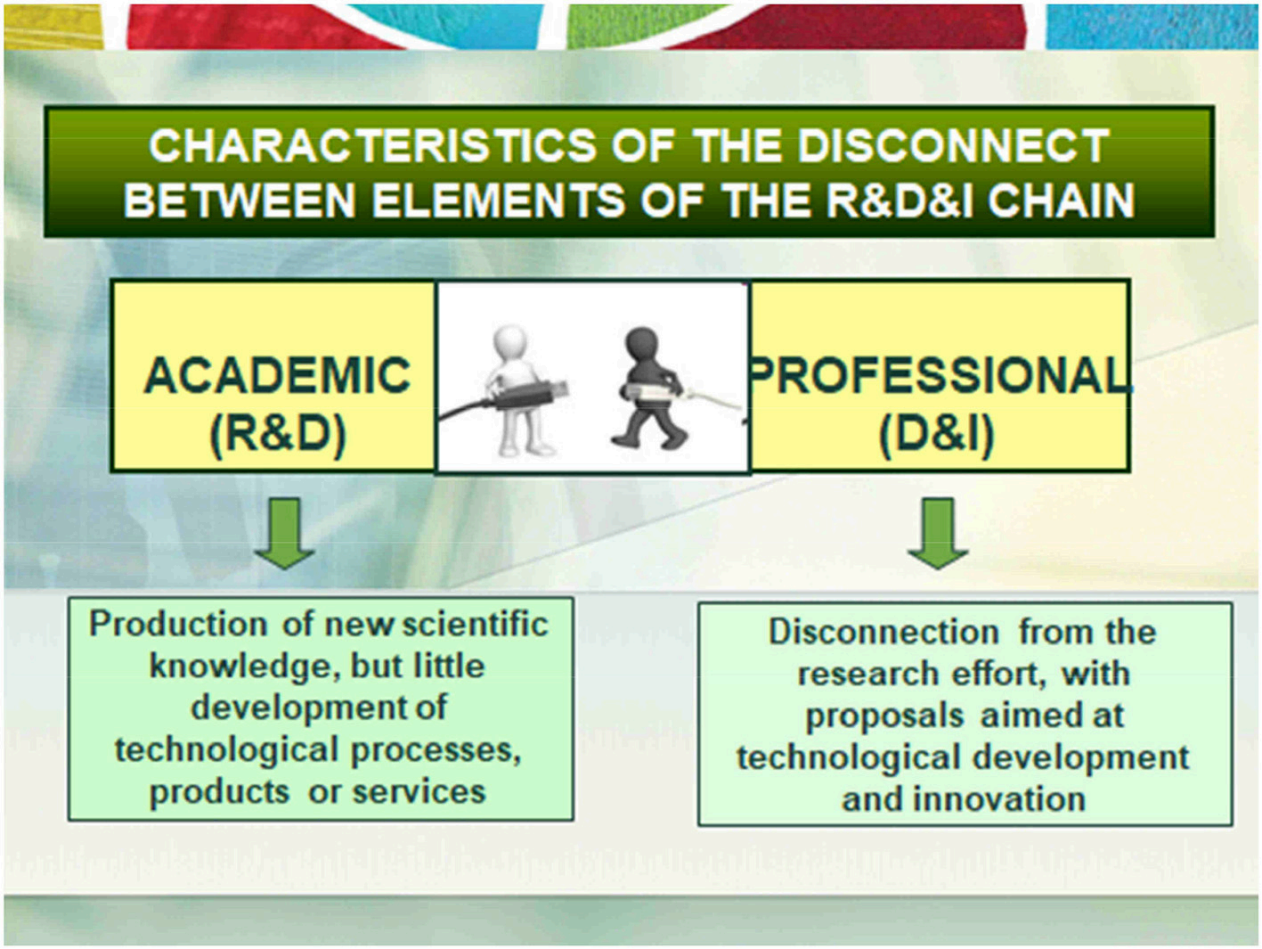
Characteristics of RD & I valor chain in Classic context.

**Figure 2:**
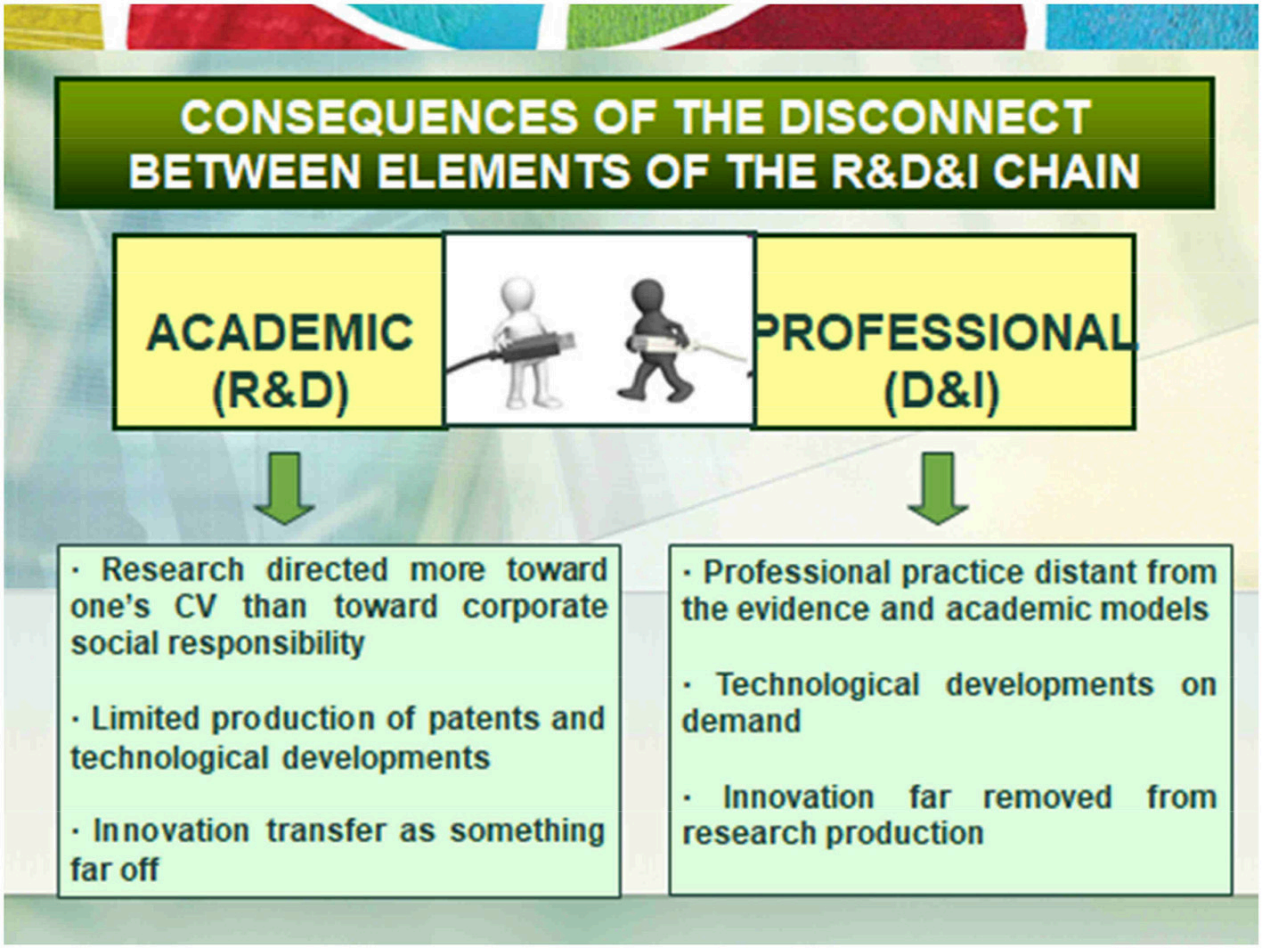
Consecuences of RD & I valor chain Classic.

1. The first consequence is the little connection between academic and professional research. The academic field has been focused on the production of knowledge, but without bringing associated technological developments and, even less, projects of entrepreneurship. For its part, the professional field was focused on making some innovations but without connection with the academic field of research and new knowledge. See Figure 1.
2. The second direct consequence has been that the academic field develops a research aimed at the production of new knowledge, preferably focused on the production of research CV and not so much on cooperative social responsibility. This implies a limited production of patents, processes, products and services of innovation and, consequently, little entrepreneurship. In the case of the professional field, interventions are carried out without being based on scientific evidence, technological developments are carried out without foundation in science or prior scientific knowledge. Therefore, it is a period of clear disconnection between the academic and professional fields. See Figure 2.

### Consequences of the current value chain

**Figure 3:**
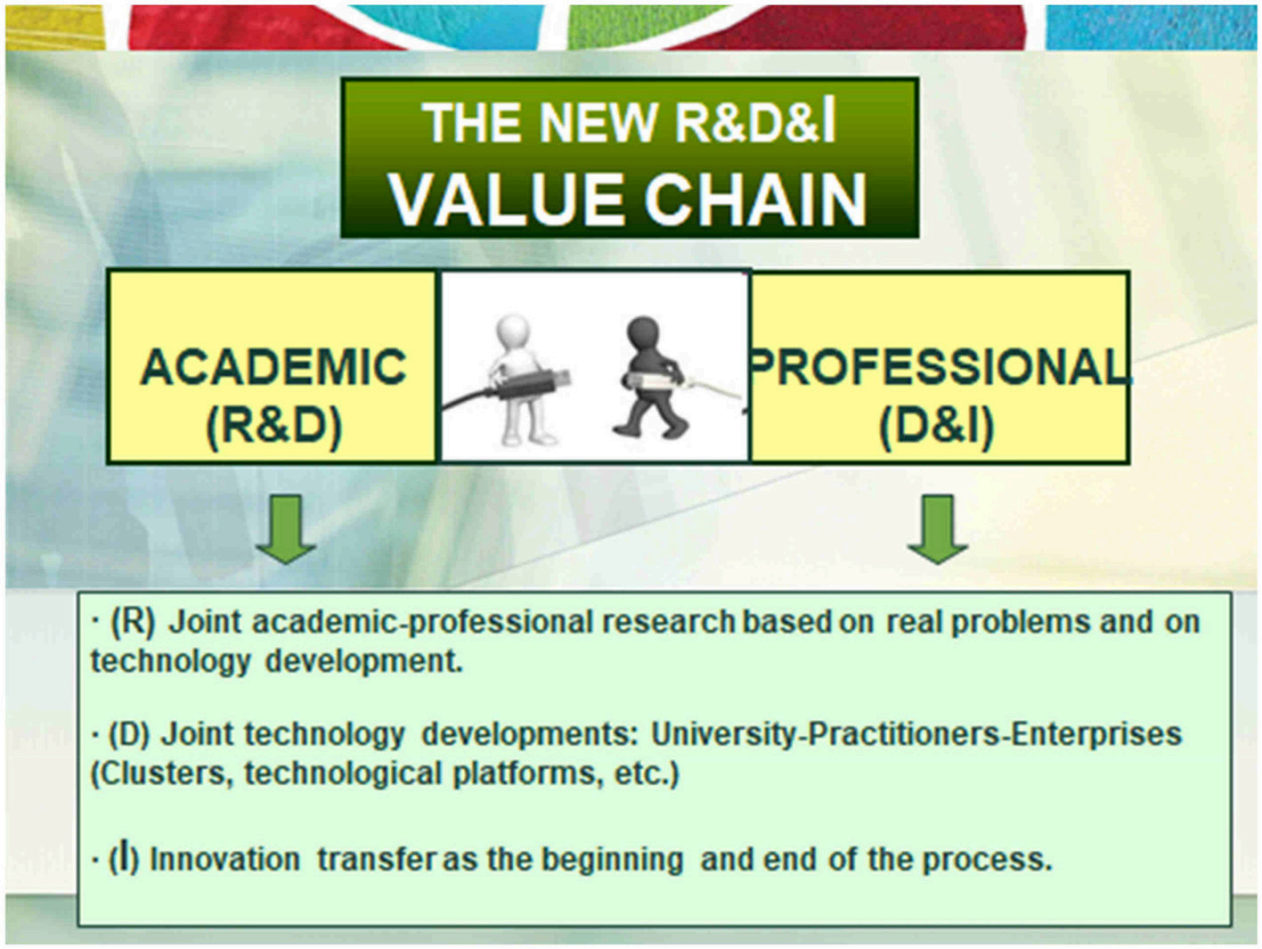
Consecuences of new R & D & I chain valor.

1. The first consequence involves a consistent and intense connection and collaboration between the academic and professional field, in both directions. The academic field provides evidence and basic and applied models, which support technological and professional development, and are even a source of creation of new entrepreneurship businesses (big data…). The professional field works in a coordinated manner with the academic, requesting new research and technological development for professional practice and entrepreneurship. In addition, it actively seeks evidence-based professional intervention, which gives the researcher an irreplaceable value of support and contribution of evidence of applied practice.
2. The second consequence of this conception of the value chain is the joint work of academic and professional researchers to achieve new technological developments and apply them to new business models to give joint answers to social demands and to problems proposed from the professional field. That is, the creation of multiprofessional teams formed by researchers, technologists, professionals and entrepreneurs who form clusters or clusters of areas such as Health and Wellbeing Technology Platforms, Technological Platforms of new ICT systems, etc. See Figure 3.

## The new RD & I value chain in educational psychology

The RD & I value chain in the sphere of Educational Psychology means recognizing that: (1) There is an important problem to be solved, preferably defined by professional practice, in reference to a process, a product or a service (*demand or need*); (2) Research actions and scientific production are to be carried out (*Research*); (3) Research actions should give rise to new technology developments in processes, products or services (*Technological Development*); (4) These developments are to be transferred and implemented in real contexts, producing innovation in professional practice (*Transfer Innovation or Entrepreneurship*). Thus, this heuristic provides for integration of *scientific or research activity* (R), professional *technology development* activity (D), and *entrepreneurship* and transfer innovation (I). In real life, the RD & I value chain must be constructed in an interdependent, coordinated fashion between the academic world (R) and the professional world (D&I). However, scientific production (R) does not always translate into technology developments (D), while in the best case scenario of professional practice, new technology tools (D) and innovations (I) are being developed, but without a clear connection to research-based scientific knowledge.

Essentially, the problem lies in an ongoing disconnection between the two contexts. Even though both the academic and professional perspectives seek to address the same problems, prospects and approaches, in many cases we find that the realities addressed are different and unconnected:

1. The *academic sphere of Educational Psychology* has focused on producing new scientific knowledge or technology developments, as well as disseminating them in formal scientific publications. However, transfer of this scientific-technological knowledge, bringing it to life in the business sphere or in society in general, has not been properly pursued. The classic academic curriculum at university has promoted the researcher profile, recognizing research as the fundamental activity.
2. In the *professional sphere of Educational Psychology*, professional practice has been pursued at some distance from the research endeavor, with few technological developments and even less innovation. A culture of professional innovation remains far from professional reality.

In today's Global Society of Knowledge, RD & I has become an engine of the economy, generating high-skilled, competitive employment in all production and service sectors. Given this panorama of the Science-Business System, an important shift is taking place in the *academic sphere* in order to promote the value chain, and innovation as an agent for strategic positioning and job creation. For example, recent proposals established a new field regarding transfer of knowledge and innovation, to be included in applications for professional advancement. Entitled “Knowledge Transfer and Innovation,” contributions in this field are valued in the following priority order:

1. Direct participation in creation of *businesses* based on the transfer of knowledge acquired through the applicant's accredited research activity. Direct participation is understood to be possession of some part of the business capital in addition to having contributed with one's work to the activity of the company.
2. *Patents in exploitation*, as demonstrated by purchasing or licensing contracts. The scope of patent protection (national, European, or through the Patent Cooperation Treaty PCT) will be taken into account. This type of contribution will also be valid if the patent has been granted by the Spanish Office of Patents and Trademarks, through the prior exam system. The number of patents applied for during the given period, regardless of whether they are in exploitation, will be given secondary consideration.
3. *Contracts with socioeconomic partners*, prompted by commercial products, innovative functional prototypes, patents in exploitation or exceptionally unique projects.
4. *Publications* drawn from work with socioeconomic partners, where commercial products, prototypes or exceptionally unique projects are described.
5. *Contributions to industrial or commercial standards* regulated by public organizations, professional societies or other entities.

Similarly, in the professional sphere, there should be a shift and a new perspective on the psychologist's practice as a player in RD & I, especially in *professional innovation*. Professional Associations should contribute significantly toward this end, with association policies that encourage and mediate RD & I—on the one hand, closely collaborating with the University, and on the other, responding to social demands, as ascertained by their professional members in their actual practice.

In the sphere of Educational Psychology, the RD & I value chain (de la Fuente and Vera, 2010) means adopting these three links as part of both *academic* and *professional* efforts in Educational Psychology. For this reason, the chain should be considered a powerful heuristic that makes it possible to integrate academic or research activity (R), professional development activity (D) and professional innovation (I). Generating scientific-technological transfer and innovation in Educational Psychology, in different educational contexts, can mean an important boost to the activity of new psychology practitioners. In the short- and mid-term progress, these results would follow: (1) Competitive positioning of psychology as a science and a professional practice, in educational contexts; (2) Production of new processes, products and innovative services in these areas; (3) Greater value given to the educational psychologist in educational contexts; (4) Creation of high-skilled jobs in this sphere.

The Psychologist -in general- and the Educational Psychologist -in particular- cannot escape this new economic and social context. The need for coordination in order to define RD & I actions must jointly concern the academic and the professional sphere. It means creating joint structures for coordinating activation of the RD & I chain, such as work being coordinated through technology-based businesses, joint definition of broad-spectrum priority research, creation of joint consortia, and creation of foundations for social purposes.

## A scientific-technological component of educational psychology, for producing technological innovation and transfer of innovation

Researchers and practicioners in the field of Educational Psychology typically explore relationships among developmental, learning, and teaching processes that are produced in formal, non-formal and informal educational contexts,. These relationships have been substantially altered by contemporary changes and phenomena that make up a new educational panorama:

1. The Information and Knowledge Society places the emphasis on these two aspects when putting together educational processes themselves, processes for constructing understanding or acquiring factual knowledge. In earlier times, factual knowledge was considered an essential element of a proper education and of being erudite. Today, such *knowing* is considered to take on three forms. *Principles* are needed for building useful knowledge that can be applied and is not inert. *Know-how* means being able to act in problem situations, applying real problem-solving skills in multiple dimensions of personhood (psychomotor, personal, social, cognitive and linguistic). *Will* involves adopting attitudes, values and habits of knowledge. Therefore, the concept of education has progressed from a merely factual view toward the idea of competencies that integrate the three levels of human knowledge.
2. Second, it is necessary to reflect on how innovation competencies can be developed in this sphere of academic and professional knowledge. There is a verified need to adjust the profile of competencies needed for successful adaptation within the Knowledge Society and Economy (Euridyce, 2005). The need for persons with training in language competencies, digital competencies, social competencies, environmental competencies, competencies in lifelong learning and in innovation, through adopting an entrepreneurial attitude, all these have been considered as basic and higher-level competencies (Lucas, 2007) to be addressed by our educational system.

The idea of *innovation and science/technology transfer* has appeared only recently in our educational and production systems. Thus, we are currently in a process of assimilating and progressively working out this personal, social and epistemological reality. In fact, the psycho-educational variables that determine this reality are yet to be defined by research: personal variables that determine an innovative and entrepreneurial attitude, educational variables that strengthen habits of innovation in science and technology, the extent to which educational and curriculum experiences encourage the choice of science and technology careers, and the level of transfer of graduates into positions requiring scientific-technological skills.

Social sciences, on the other hand, have had little orientation toward innovation and science or technology transfer. In the social realm, educational work is considered to be a services activity, not connected to the production sector. This contributes to the idea that there is little need for generating new processes, products and services. Only the arrival of ICTs has brought about a significant move toward such innovation. To encourage the necessary paradigm shift that produces meaningful innovation and transfer in today's information- and knowledge-based society, we look to progress in Educational Psychology *Research* (R). Besides leading to new knowledge, it can produce new *technological Developments* (D) that then take shape in new *processes, products and services* (I) with direct application (Education & Psychology I+D+i, 2008).

In addition, we would begin experiences and training in the RD & I value chain with new generations of psychologists. The new Bachelor's and Master's degree programs are an opportunity to introduce training processes that establish innovation as an essential working tool. In particular, Master's degrees should work on the innovation and entrepreneurship competency, in each of the class subjects, as an essential process for decision making in academia and professional practice, so as to create new work opportunities and new positioning as a psychologist within the labor market (de la Fuente et al., 2012). Medium- and long-term results of such a philosophy, for the science and in the profession, would include:

1. Better strategic positioning of the profession and of professionals within the Information and Knowledge Society: There are emerging professional profiles of scientists, technologists and practitioners that are related to psychology; these can lead to recognition for Educational Psychology and make it competitive as a science and profession.
2. Creation of competitive processes, products and services with high innovative value: We can carry out innovative proposals in Educational Psychology for identifying problems, assessing and intervening, with new parameters based on the use of ICTs (European Communities, 2006).
3. Specialization of new professionals in the RD & I value chain: The training of new professionals, through Master's and Doctoral programs that are properly integrated in the RD & I value chain would mean redefining relations between the science and the profession.

This innovation proposal continues to be a challenge for *academia* and for the *profession*, in the interest of creating skilled positions with a new professional slant, in organizations and institutions that work across the entire domain of Educational Psychology. Toward this end, there is a pressing need for close collaboration between academics and practitioners:

1. New conception of the task of research and professional practice.
2. New conception of Master's programs and RD & I Projects
3. New conception of Doctoral programs and of doctoral dissertations
4. New collaboration structures between academic and professional spheres: RD & I Departments, Technology-Based Enterprises.

### The news conception of R & D & I projects in educational psychology

In the context of the most recent vision to R & D & I value chain, the actual conception of R & D Project has three components (Sterlacchini, 2008): (1) Research, (2) Technological Development, and (3) Trasfer Innovation or Entrepreneurship:

1. *Research component*. Investigate the relationships between variables that explain stress behaviors during university learning-teaching process, and their effect on performance, with special attention to coping strategies (*Scientific Research*).
2. *Development component* of new ICT technology of process, product of servirces, which provide a response to real problems in profesional practice (*Technological Developments*).
3. *Transfer and exploit* this innovation through the services sector, especially through interested Technology-Based Enterprises (*Transfer Innovation or Entrepreneurship*). Based on recent technological systems, these represent innovation that can be transferred to the professional and business sector. The industrial, technological and professional sectors can solicit these. The innovation transfer, taking place through science and technology transfer seminars, RD & I Departments and TBEs (spin-off) enterprises.

### Components and functions of an RD & I department in educational psychology organizations and services

Unlike other professional fields where the RD & I Department is an unquestionable reality (cf. experimental science and technology), this idea has yet to be represented and developed in the professional sphere of the Social Sciences. Today's reality is increasingly competitive. If we want to be leading societies in the production of knowledge, products and services, we must not fail to adopt an innovative spirit. We are immersed in a scientific-technological system where we are funding innovations that will be in the market 10 years from now. It is evident, then, that decisions made in the present will give shape to our future, and will or will not make us competent. For this reason we must not take a passive posture, but we must be active and adopt the changing trends of the Knowledge Society. Development of RD & I Departments can help us rise to the challenge of this context of change.

Although Psychology itself has coined expressions that would emphasize a scientific-technological viewpoint (for example, behavioral engineering), the reality is that few psychologists consider establishing RD & I Departments in the organizations where they exercise professional influence, whether educational or other. For this concept to materialize, a new, overarching area would be created in both professional institutions and educational organizations. Whether an RD & I Department as such, or some area that cuts across the others (de la Fuente, 2010), its mission includes the actual implementation of the RD & I chain in the educational organization, and incentivizing innovation activities (de la Fuente and Zapata, 2012). Such innovation would not only relate to teaching, but to innovation in different education and psychology programs, whether pertaining to the organization, assessment, analysis of information generated by the organization itself, cost-benefit analyses, strategic decision-making processes, etc.

The *RD & I Dept*. with its cross-cutting nature, ought to become central to the academic and professional practice of psychology in this century. In the case of the RD & I Department, its principal objective would be research support and professional support for different sectors of education or businesses involved in this field. Such support would contribute to the realization of individual projects or collaborative projects with national or international institutions, as well as facilitate access to possible funding sources. This department would offer up-to-date information about RD & I incentives and available assistance, in addition to supporting the phase of project definition and preparing applications for assistance from each of the different public RD & I programs. Similarly, it would facilitate the search for partners, in any geographic area, that are best suited to the project (universities, SMEs, users, etc.). With these issues in mind, we define the dimensions of RD & I for Educational and School Psychology. This proposal is articuled through the following Working Topics, as previosly descrived (de la Fuente and López, 2007; de la Fuente and Zapata, 2012). See Table 2.

**Table 2:** Subareas, justification, competencies and services of RD & I Department in Educatinal Psychology.

**Subarea**	**Justification**	**Competencies**		
Educational Psychology Research	Need for professionals for the study of processes or products of the Organization as a whole, or of the Guidance Department in particular. Professional competencies required in making research decisions (de la Fuente and Justicia, 2018).	1. Adopt theoretical models for Applied Research on Processes and Products (Professional Assessment and Intervention) in the chosen problem area. 2. Do bibliographic searches and use decision-making criteria in their selections. 3. Draw up Research Designs for their own real-life context. 4. Apply Instrument Models and Research or Assessment Tools to their own issues and real-life context. 5. Execution of the Research Design and of Professional Intervention. 6. Data Analysis and Processing of the above. 7. Draw Conclusions. 8. Draft the Research Report. 9. Publication and/or Communication of Results (de la Fuente and López, 2007). 10. Familiarity with recent professional research.		
Technology Development in Educational Psychology	Re-conceptualizing Educational Psychology as an *essential agent in pursuing quality and developing new scientific-technological products* for professional use, especially those pertaining to assessment and intervention. Development of ICTs applied to professional practice is especially valuable.	1. Detect needs in educational practice and in the guidance role itself. 2. Develop or adopt existing models and tools that (1) are based on evidence from professional practice and research projects, and (2) that respond effectively to significant problems, typical of professional practice. 3. Generate synergy through connecting scientific-technological development from the University with its application to professional knowledge and issues. 4. Propose tools and technology developments in ICT formats that can respond to school psychology problems. 5. Create R&D consortia for collaboration between the University and Professional Institutions.		
Transfer Innovation in Educational Psychology	Educational Guidance Department as a catalyst to innovation in any field of educational practice. Increasing quality and educational action in any activity, but especially in intervention for prevention of problems, or in promoting experiences with educational innovation.	1. Innovating in the practice of education and school psychology, based on experiences and tools that have been researched and validated. 2. Encouraging innovation as a tool for professional and personal growth, generating scientific-technological contexts within the field of professional practice. 3. Integrating and generalizing ICTs in the field of education, and in psychological advising and guidance at school (Calik et al., 2017).		
**Subarea**	**Services and tools: mainstream teaching**	**Services and tools: attention to diversity and special educational needs**	**Services and tools: academic and vocational guidance**	**Example of action steps**
Educational Psychology Research	Evaluation, investigation and improvement of the processes of development, learning and teaching.	Evaluation and investigation of learning problems, and of developmental and learning disabilities.	Evaluation and investigation in Academic and Vocational guidance.	1. Conceptualize research for screening, assessment and intervention in educational psychology issues (de la Fuente and Justicia, 2018). 2. Articulate and execute applied research projects. 3. Request research projects, in collaboration with university researchers and institutions. 4. Apply important scientific-technological advances gained from the evidence of regional, national and international R&D projects. 5. Present scientific-professional reports to the community, institution or organization, underscoring the effects and profitability of the action steps that were taken.
Technology Development in Educational Psychology	Development and validation of programs and tools for assessment and intervention in the processes of development, learning and teaching.	Development and validation of programs and tools for assessment and intervention in learning problems, and in developmental and learning disabilities.	Development and validation of programs and tools for assessment and intervention in academic and vocational guidance.	1. Propose the development of utilities to science and technology organizations and businesses in the sector. 2. Participate in developing and validating these utilities. 3. Promote science and technology entrepreneurship among education professionals and educational and school psychologists. 4. Collaborate in the design and development of new applications and knowledge using ICTs, in the field of education and guidance. 5. Create new tools for assessment, intervention and organization of information and knowledge in this professional field.
Transfer Innovation in Educational Psychology	Innovation in the use of ICTs, assessment tools and programs that intervene in the processes of development, learning and teaching.	Innovation in the use of ICTs, assessment tools and programs that intervene in the problems of learning, developmental disorders and learning disorders.	Innovation in the use of ICTs, assessment tools and programs that intervene in Academic and Vocational Guidance.	1. Innovate in the use of ICTs in different areas of advising (Newman et al., 2017). 2. Implement virtual communities. 3. Incorporate ICTs in administration of processes and products from the Guidance Dept. 4. Collaborate online with academic and professional consultants and experts. 5. Innovate on a daily basis in the practice of educational and school psychology.

This approach would have a number of consequences: (1) Demand for new professional profiles, for educational psychologists that specialize in research, technological development and applied innovation. (2) Exploitation of resources that are presently underutilized, such as the information generated by the organization itself. (3) New technological developments, in ICT formats, for assessment and intervention (Tavassoli and Carbonara, 2014). (4) Innovation in Educational Psychology becomes a reality (Caro-Vargas, 2017).

The RD & I value chain, in the shape of an RD & I Department in educational organizations, or any organization where psychologists are involved, would generate new professional activity, improve the practice of Educational Psychology and create demand for qualified professionals to fill these posts. We as psychologists have the conceptual, methodological and applied training in order to make this idea a reality (de la Fuente and Zapata, 2012).

### Technology-based companies (spin-off) as a tool for entrepreneurship and transfer of innovation in educational psychology

The RD & I value chain can mean an advantage to the different activities of academics, research, and professional practice, with respect to processes, products and services that are generated in the sphere of psychology and education. Several examples are presented as to how the RD & I chain can help to improve actions. The RD & I chain is exemplified in the development of new processes, products and services, in the *technology-based business* itself, as a practical example of the paradigm of innovation transfer and psychological entrepreneurship (Matlay, 2008; Schaltegger and Wagner, 2008; Pyka and Prettner, 2018).

The lack of an integrated RD & I value chain in the different scientific and professional tasks from the sphere of Educational Psychology has had several practical consequencies. First, it has given rise to excessive specialization in one link of the chain, focusing on one end or the other, and losing sight of the chain itself. Second, the different tasks (research, development, innovation) are represented in isolation and with unequal value. In a classic approach, the researcher who carries out projects sees no need to move on toward later technological developments or the transfer of his/her research to new innovative processes, products or services within the professional market. Similarly, the psychology practitioner is not always sensitive to effects that innovation can produce in professional activity, considering that research and scientific-technological development are far removed from his or her immediate professional demands, and the RD & I chain has little to do with his/her reality.

The classic conceptual representation of theory vs. practice has led to gaps in the relationship between the tasks of research (R), scientific-technological development (D), and professional innovation (I). Theory cannot always be prior to and disconnected from practice, nor vice versa. We should adopt the view that all Educational Psychology work is located along some point of this chain, and that should prompt us to coordinate with its other elements (NESTA, 2008). There are different examples of how the RD & I chain can help improve the quality of actions taken in Educational Psychology. In Spain, competitive improvements in the RD & I chain can be pursued through public bidding for *Research Projects* (D'Ambrosio et al., 2016).

## Conclusions

Not adopting the RD & I value chain concept may have severe practical consequences in scientific and professional tasks. In the first place, the focus is placed on one or another of the three endeavors, overlooking the chain itself. Second, the tasks of research, development and innovation are represented without any connection. In the classic style, the researcher who carries out projects does not see the need to progress toward follow-on technological developments, and their transfer to the market in the form of innovation. Conversely, the professional who wishes to innovate or reinvent his or her professional activity, sees no need to begin from the research, which is perceived as distant from reality (Ertuna and Gurel, 2011).

This perceived relationship between theory and practice, now a foregone conclusion, has led us to make errors in defining research and professional work. Theory need not always be prior to and disconnected from practice, nor is the converse necessarily required. All of us who work in Educational Psychology ought to recognize that we are placed at some point in this chain, and that fact should prompt us to coordinate with other elements on the same chain.

The only viable solution for raising the value of *Educational Psychology* practice is a significant move, from both ends of RD & I chain, toward connecting the links:

1. From the *academic sphere*, academic researchers (R) must become concerned with the development and production of new processes, products and services (D), and finally, that these be transferred in order to implement innovation in real professional contexts (I).
2. From the *professional sphere*, psychology practitioners who seek to innovate in their practice (I) must make professional demands for creation of new processes, products and services (D), based on the academic knowledge gained from research (R) (Wang et al., 2013).

This coordinated, joint work requires new actions and new structures for it to materialize on a permanent basis. Creation of cooperative agreements or scientific-technological consortia for the purpose of promoting joint RD & I between the University and Professional Associations can be a tool to help new professionals position themselves in the twenty first century Knowledge Society, adopting new professional profiles and activities in Educational Psychology (European Commission, 2006). Realization of this idea would be profitable for both research and professional practice, making a positive difference in the processes, products and services that are produced in the educational psychology sphere and that form part of the professional profile, and education for entrepreneurship in psychologists (Pittaway and Cope, 2007; Oosterbeek et al., 2010). This approach means unequivocably bridging the gap between science and profession, between researchers and professionals, in order to jointly redefine the big challenges that face the science and the profession (Walter et al., 2013). Only in this way can Psychology -and especially Educational Psychology- position itself strategically in the present-day international context of Science and Innovation (de la Fuente and Vera, 2010; Subramanian et al., 2016), alongside other social sciences, education and healthcare (Seelos and Mair, 2005; European Commission, 2014; Ferguson, 2016). At present, steps are being taken in this direction (de la Fuente et al., 2018).

## Author contributions

All authors listed have made a substantial, direct and intellectual contribution to the work, and approved it for publication.

### Conflict of interest statement

The authors declare that the research was conducted in the absence of any commercial or financial relationships that could be construed as a potential conflict of interest. The handling Editor declared a shared affiliation, though no other collaboration, with one of the authors DK.
